# SARS-CoV-2 Omicron BA.2.75 Variant May Be Much More Infective than Preexisting Variants Based on In Silico Model

**DOI:** 10.3390/microorganisms10102090

**Published:** 2022-10-21

**Authors:** Aki Sugano, Yutaka Takaoka, Haruyuki Kataguchi, Mika Ohta, Shigemi Kimura, Masatake Araki, Yoshitomo Morinaga, Yoshihiro Yamamoto

**Affiliations:** 1 Center for Clinical Research, Toyama University Hospital, Toyama 930-0194, Japan; 2 Department of Medical Systems, Kobe University Graduate School of Medicine, Kobe 650-0017, Hyogo, Japan; 3 Department of Computational Drug Design and Mathematical Medicine, Toyama University Graduate School of Medicine and Pharmaceutical Sciences, Toyama 930-0194, Japan; 4 Data Science Center for Medicine and Hospital Management, Toyama University Hospital, Toyama 930-0194, Japan; 5 Center for Advanced Antibody Drug Development, University of Toyama, Toyama 930-0194, Japan; 6 Life Science Institute, Kobe Tokiwa University, Kobe 653-0838, Hyogo, Japan; 7 Division of Genomics, Institute of Resource Development and Analysis, Kumamoto University, Kumamoto 860-0811, Japan; 8 Department of Microbiology, Toyama University Graduate School of Medicine and Pharmaceutical Sciences, Toyama 930-0194, Japan; 9 Department of Clinical Infectious Diseases, Toyama University Graduate School of Medicine and Pharmaceutical Sciences, Toyama 930-0194, Japan

**Keywords:** SARS-CoV-2, COVID-19, spike protein, evolutionary distance, docking affinity

## Abstract

Previously, we developed a mathematical model via molecular simulation analysis to predict the infectivity of six SARS-CoV-2 variants. In this report, we aimed to predict the relative risk of the recent new variants of SARS-CoV-2 based on our previous research. We subjected Omicron BA.4/5 and BA.2.75 variants of SARS-CoV-2 to the analysis to determine the evolutionary distance of the spike protein gene (S gene) of the variants from the Wuhan variant so as to appreciate the changes in the spike protein. We performed molecular docking simulation analyses of the spike proteins with human angiotensin-converting enzyme 2 (ACE2) to understand the docking affinities of these variants. We then compared the evolutionary distances and the docking affinities of these variants with those of the variants that we had analyzed in our previous research. As a result, BA.2.75 has both the highest docking affinity (ratio per Wuhan variant) and the longest evolutionary distance of the S gene from the Wuhan variant. These results suggest that BA.2.75 infection can spread farther than can infections of preexisting variants.

## 1. Introduction

In Japan, as of July 2022, infection with the Omicron BA.5 variant of SARS-CoV-2 has become an epidemic disease. In addition, Omicron BA.2.75 was discovered and is thought to present a particular risk inasmuch as it may cause a coming epidemic. We previously constructed a mathematical model to predict the infectivity of SARS-CoV-2 variants—Alpha, Beta, Gamma, Delta, Omicron BA.1, and BA.2 as a ratio per Wuhan variant [1]. In this research, we report the predicted risks for Omicron BA.4/5 and BA.2.75, which were recently recognized as being causes of epidemic diseases. For this purpose, we utilized the analyses of the evolutionary distance and the docking simulation that we established in our previous research [1].

## 2. Materials and Methods

### Determination of the Absolute Evolutionary Distances between the Wuhan Variant and Variant Spike Protein Genes (S Genes), and Docking Affinities of the Different Spike Proteins with ACE2

We analyzed the absolute evolutionary distances of the S gene from the Wuhan variant for variants —Alpha, Beta, Gamma, Delta, Omicron BA.1, BA.2, BA.4/5, and BA.2.75 via the ClustalW program [2] and FastTree program [3]. We obtained the sequences of the S gene by searching NCBI (MN908947 for Wuhan, OW519813 for Alpha, OM791325 for BA.1) [4] or the EpiCoV database of GISAID for the complete sequence of the S gene (EPI_ISL_5142896 for Beta, EPI_ISL_14534452 for Gamma, EPI_ISL_4572746 for Delta, EPI_ISL_13580480 for BA.2, EPI_ISL_13304903 for BA.4/5, and EPI_ISL_14572678 for BA.2.75) [5,6].

We used docking simulation to investigate the docking affinity of the receptor binding domain (RBD) of each variant spike protein with ACE2 [1]. We defined the docking affinity here as the most stable score in the docking results with correct binding mode.

We obtained the information for the amino acid substitutions of the spike proteins from the CoVariants website, which classifies the variants according to Nextstrain clades [7,8]. We then used the amino acid sequences for the three-dimensional structures for the docking simulation. BA.4 and BA.5 have identical spike proteins, and thus we grouped the sequence of their S genes or their amino acid sequences together in this research.

## 3. Results

### Absolute Evolutionary Distances for S Gene Variants and Results of Docking of the RBD with ACE2 Protein

Table 1 shows the absolute evolutionary distances of the S gene between the Wuhan variant and each of the other variants, as well as the docking affinities of the RBD of the spike protein with ACE2 (ratio per Wuhan variant), which we determined from the docking simulation. The variants with longer evolutionary distances from the Wuhan variant had a tendency toward causing more epidemics. The Omicron BA.2.75 variant had the highest docking affinity of the spike protein with the ACE2 protein.

In comparison with BA.2, BA.4/5 had a longer S gene evolutionary distance but showed a lower docking affinity. BA.2.75 had not only a longer evolutionary distance but also a higher docking affinity than BA.2. 

## 4. Discussion

For this report, we analyzed the evolutionary distance of the S gene and the docking affinity with ACE2 of the recent new Omicron variants, BA.4/5 and BA.2.75. We focused on only the S gene sequence to calculate the evolutionary distance and did not use the genome sequence, which is used for the phylogeny provided by Nextstrain [7] because it is the spike protein that binds to ACE2 on human cells.

Our analyses are based on the following two factors, which play important roles in the infectivity of the virus variants: (1) the ability of the virus to enter human cells, which is the primary stage for virus infection, and (2) the effect of a neutralizing antibody in humans or the effect of vaccines. The first is demonstrated by the docking affinity of each RBD in the spike protein with ACE2, that is, which docking affinity is greater. The second is shown by the evolutionary distance of the S gene from the Wuhan variant: the longer the distance is, the weaker the effect of vaccines. It is based on our previous findings of the correlation between S gene evolutionary distance and the infectivity of SARS-CoV-2 variants [1], and the currently available vaccines were developed on the basis of the Wuhan variant [9]. However, further stages of virus infection, such as the risk for exacerbation and the growth rate of SARS-CoV-2, cannot be appreciated via the two factors.

Omicron BA.2.75 showed the highest docking affinity of the spike protein with the ACE2 protein compared with the other seven variants (ratio per Wuhan variant). In addition, the S gene evolutionary distance of BA.2.75 from the Wuhan variant was the longest of the variants. Although the results are based on in silico model, our simulation experiments and analyses are based on actual data, including the ones from in vivo or in vitro studies as described in our previous research for the details of molecular simulations [1]. Our analyses revealed that BA.2.75 has a superior ability to enter human cells, and also, the current vaccines can be less effective against this variant. These results suggest that the BA.2.75 infection can spread farther than can infections of preexisting variants. In addition, our results indicate the need for great caution in managing BA.2.75 because the number of severely ill patients or sufferers will be increased along with the increased number of infected individuals, even if this variant has a low risk for exacerbation.

## 5. Conclusions

We demonstrated here that the Omicron BA.2.75 variant of SARS-CoV-2 has the longest results with regard to the evolutionary distance of the S gene from the Wuhan variant and the highest results of the docking simulation for spike protein with ACE2. Our results based on in silico model indicate that Omicron BA.2.75 poses a greater risk to global health than other variants and that we must pay close attention to the Omicron BA.2.75 infection trends.

## Figures and Tables

**Table 1:** The evolutionary distance of the S gene and the docking affinity of the spike protein with ACE2 (ratio per Wuhan variant).

Variants	Wuhan	Alpha	Beta	Gamma	Delta	Omicron BA.1	Omicron BA.2	Omicron BA.4/5	Omicron BA.2.75
Pango Lineage	B	B.1.1.7	B.1.351	P.1	B.1.617.2	B.1.1.529/BA.1	B.1.1.529/BA.2	B.1.1.529/BA.4/5	B.1.1.529/BA.2.75
Absolute evolutionary distance of the S gene (from Wuhan) × 10^−3^	-	2.07	2.07	3.55	3.26	10.72	8.31	9.21	10.99
Docking affinity (ratio per Wuhan)	1	1.18	1.23	1.31	2.10	1.55	2.46	2.15	2.90

## Data Availability

Data that support the findings of this study are available from the corresponding author upon reasonable request, except publicly available data sources.

## Abbreviations

Spike protein gene, S gene; angiotensin-converting enzyme 2, ACE2; receptor binding domain, RBD.
